# An *AGAMOUS*-like factor is associated with the origin of two domesticated varieties in *Cymbidium sinense* (Orchidaceae)

**DOI:** 10.1038/s41438-018-0052-z

**Published:** 2018-09-01

**Authors:** Shihao Su, Xiaoyu Shao, Changfa Zhu, Jiayin Xu, Yuhuan Tang, Da Luo, Xia Huang

**Affiliations:** 1 State Key Laboratory of Biocontrol and Guangdong Key Laboratory of Plant Resources, School of Life Sciences, Sun Yat-sen University, Guangzhou, 510275 China; 2 Institute of Transformative Bio-Molecules (WPI-ITbM), Nagoya University, Furo-cho, Chikusa-ku, Nagoya, 464-8601 Aichi Japan

## Abstract

*Cymbidium* has been artificially domesticated for centuries in Asia, which produced numerous cultivated varieties. Flowers with stamenoid tepals or those with multiple tepals have been found in different species of *Cymbidium*; however, the molecular basis controlling the formation of these phenotypes is still largely unknown. Previous work demonstrated that *AGAMOUS/AG* lineage MADS genes function in floral meristem determinacy as well as in reproductive organs development in both dicots and monocots, indicating a possible relationship with the origin of two flower varieties in *Cymbidium*. Here, we characterized and analyzed two *AG* lineage paralogues, *CsAG1* and *CsAG2*, from *Cymbidium sinense*, both of which were highly expressed in the gynostemium column of a standard *C. sinense*. Interestingly, we detected ectopic expression of *CsAG1* rather than *CsAG2* in all floral organs of a stamenoid-tepal variety and significant down-regulation of *CsAG1* in a variety with multiple tepals. Over-expression of *CsAG1* in wild type *Arabidopsis* resulted in petal-to-stamen homeotic conversion, suggesting a conserved C-function of CsAG1 in the development of *Cymbidium* flower. Altogether, our results supported a hypothesis that disruption of a single *AG*-like factor would be associated with the formation of two domesticated varieties in *C. sinense*.

## Floral development: How the plum blossom orchid got its shape

A single gene underlies the remarkable differences between the flowers of two varieties of the popular orchid *Cymbidium sinense*. Orchid flowers are highly specialized, with many unique flower structures. Although the genetics of flower development have been well-studied, they are not as well understood in orchids. Xia Huang at Sun Yat-sen University in China and coworkers studied the genetics underlying differences between a standard *C. sinense* flower and a flower of the “Ling-Nan-Da-Mei” or plum-blossom variety, in which the tepals (the outer petal-like structures) strongly resemble stamens (which produce pollen). They found that in the “Ling-Nan-Da-Mei” flowers, a gene that controls stamen development is also switched on in the tepals, giving them a more stamen-like shape. Studying how genetic changes affect flower form can illuminate how so many different types of flowers evolved.

## Introduction

The *Cymbidium* spp. have been cultivated for more than ten centuries in Asia, including China, Japan, Korea, and many other places^1,2^. After hundreds of years of domestication, numerous varieties with diversification in inflorescence architecture, leaf or flower color, fragrance as well as tepal shape have been produced. Among these varieties, flowers with stamenoid tepals or those with multiple tepals widely exist in different *Cymbidium* species such as *C. goeringii*, *C. faberi*, and *C. sinense*, which were documented in an ancient Chinese Orchidology book named “Nan-Zhong-You-Fang-Lu” in around 1412 Common Era.

**Fig. 1:**
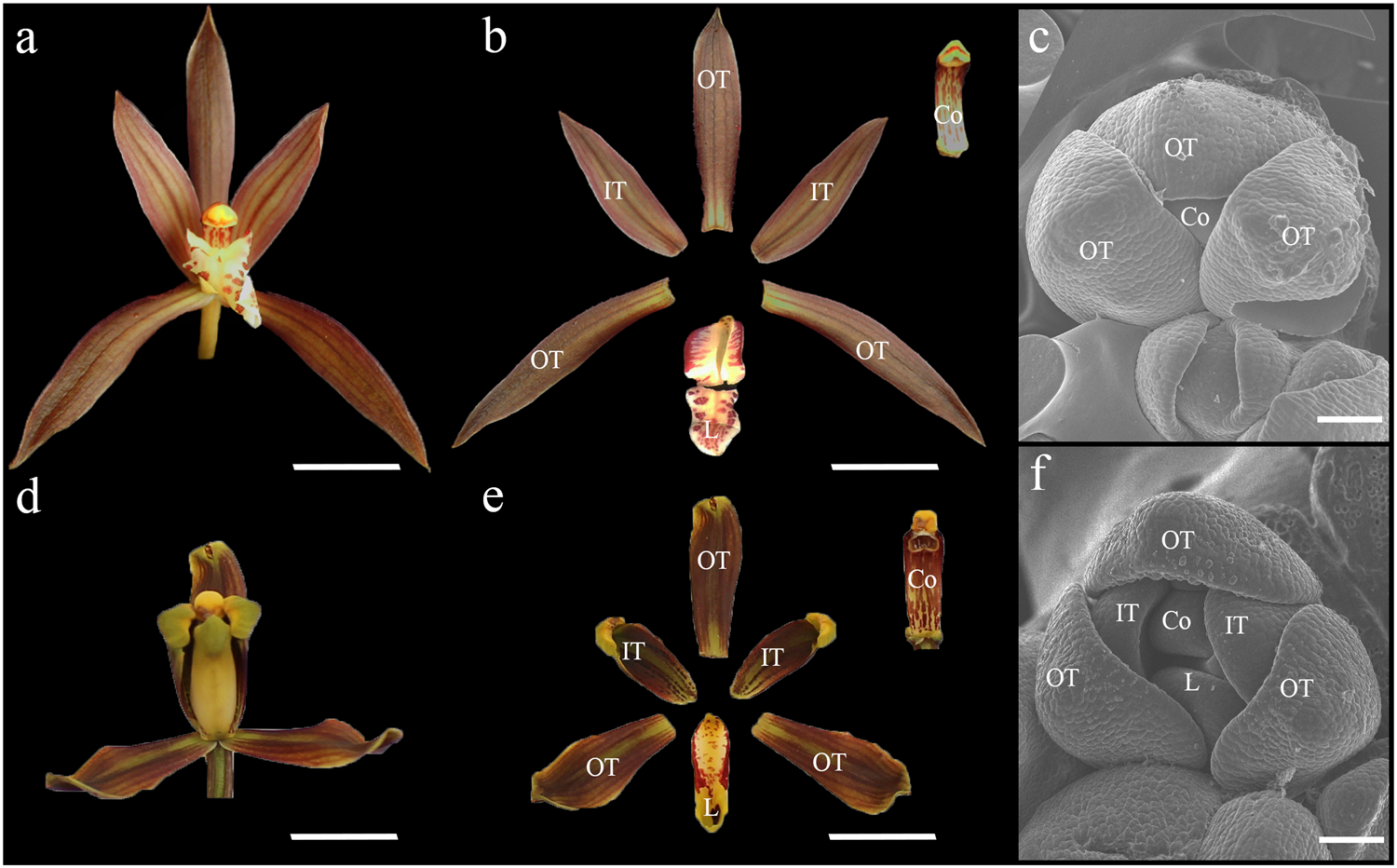
Floral phenotypes of the standard and stamenoid-tepal *Cymbidium sinense*. A mature standard (**a**) and stamenoid-tepal (**d**) *C. sinense* flower; a dissected standard (**b**) and stamenoid-tepal (**e**) *C. sinense* flower; OT, outer tepal; IT, inner tepal; L, lip; Co, column; bar = 10 mm. Scanning electron microscopic photographs of a developing standard (**c**) and stamenoid-tepal (**f**) flower; bar = 100 μm

A standard *C. sinense* flower (CsWT) possesses three distinct whorls of floral organs (Fig. 1a). The outermost whorl consists of three long outer tepals, also known as sepals; the second whorl consists of three shorter inner tepals, also known as petals, with the dorsal one differentiated into a spotted showy lip; the innermost whorl is the reproductive structure called gynostemium or column, where both male and female reproductive organs are fused together into a single column (Fig. 1b). Due to the highly specialized floral organs and numbers of cultivated varieties, *C. sinense* becomes an ideal material to study the origin of particular floral forms^3^.

MADS-box genes containing a highly conserved M domain have been widely recruited in the flower developmental processes, among which the AGAMOUS/AG lineage is involved in floral meristem determinacy and confers the identity of reproductive floral organs including stamens and carpels^4–9^. Phylogenetic analyses of different *AG*-like sequences demonstrated that gene duplications occurred extensively during the evolution of this subfamily^10^. In core eudicots, the AG lineage can be further divided into two sub-clades including the euAG and PLENA/PLE. Within monocots and other basal eudicots, the evolutionary scenario of AG lineage factors is still obscure, although multiple gene duplication events have been observed^6,10^.

The function of AG lineage factors has been very well characterized in the core eudicots^7–13^. In *Arabidopsis thaliana*, mutation in the *euAG* sub-clade member *AG* exhibits loss of stamen and carpel identity, with defects in floral meristem determinacy that result in the development of another flower in place of the carpel^9,13^. Further, AG interacts antagonistically with APETALA2/AP2, another floral homeotic gene regulating normal development of the first sepal whorl and the second petal whorl^8,9^. AP2 restricts the expression of *AG* gene in the two inner whorls, or counteracts AG activity in the center of flower^8,9,14^. In snapdragon (*Antirrhinum majus*), mutation in *euAG* sub-clade *FARINELLI/FAR* produces normal flowers with partially male-sterile^12^; while in petunia (*Petunia hybrida*), down regulation of *euAG* sub-clade gene *PETUNIA MADS BOX GENE3/PMADS3* results in mild abnormalities in reproductive organ development^11^.

The *PLE* sub-clade *AG* factors work redundantly with the *euAG* sub-clade members. Two *PLE* sub-clade genes from *Arabidopsis*, *SHATTERPROOF/SHP1* and *SHATTERPROOF2/SHP2*, exhibit partly redundant function to *AG*, responsible for stamen and carpel development^15^. In snapdragon, reproductive organs of *ple-1* (*PLE* sub-clade) mutant are converted into perianth organs, showing severely developmental defects when compared to the *far* (*euAG* sub-clade) mutant^12^. Genetic analysis suggests that *FAR* negatively regulates the expression of *PLE*^12,16^. In petunia, *PLE* sub-clade *FLORAL BINDING PROTEIN6/FBP6* and *euAG* sub-clade *PMADS3* have largely overlapping in function, determining reproductive organ identity as well as floral determinacy^11^.

In grasses, such as rice (*Oryza sativa*) and maize (*Zea mays*), the *AG*-like members have also been analyzed. In rice, two members, OsMADS3 and OsMADS58, belong to the AG lineage. In the knockout line of *OsMADS3*, stamens transform into lodicules and ectopic lodicules develop in the second whorl of floral organs^5^. In the *osmads3 osmads58* double mutant, the reproductive organ identity is completely lost together with the loss of floral meristem determinacy, and massive lodicules-like structures appear in the third and fourth whorls^17^. In maize, there are four members in AG lineage, including ZAG1, ZAG2, ZMM2 and ZMM23. *ZAG1* is highly expressed in stamen and carpel primordia; however, those mutants show a loss of floral meristem determinacy, rather than serious defects in reproductive organ identity^4,18^. The different expression pattern of these genes favors subfunctionalized behaviors of maize AG-like factors in regulating stamen, tassel, and carpel development of male and female flowers^4,6,18–20^.

Several AG lineage factors have been isolated from Orchidaceae species^21–26^. In *Dendrobium crumenatum*, the putative C function gene *DcOAG1* is highly expressed in all the floral organs, which leads to *ap2*-like phenotypes when ectopically expressed in *Arabidopsis*^23^. In the orchid *Erycina pusilla*, three *AG* lineage factors *EpMADS20/21/22* are all strongly expressed in the column whorl indicating possible functional redundancy in male and female reproductive organ development^23^. In a more closed relative of *C. sinense*, *C. ensifolium*, duplicated AG lineage genes denoted *CeMADS1/2* have been characterized^24^. In the *multitepal* mutant, whose column has been centripetally replaced by numerous tepal-like structures, the normal expression of *CeMADS1* rather than *CeMADS2* is disrupted^24^. Interestingly, in the *gylp* mutant from *Phalaenopsis equestris*, whose two inner tepals change into gynostemium-like structures, an *AG* lineage gene, *PeMADS1*, is ectopically expressed in the gynostemium-like tepals^26^. All these findings give a strong indication that defects in AG-like factors would have occurred in the origin of the stamenoid-tepal and multi-tepal varieties in *C. sinense*.

In this study, we have isolated two AG lineage paralogs from *C. sinense*, and phylogenetically analyzed the evolution of AG lineage factors in Orchidaceae. We observed the floral developmental abnormalities between the standard and stamenoid-tepal variety in the early floral developmental stage. By detecting the expression patterns of these factors, we found that *CsAG1* but not *CsAG2* is ectopically expressed in all floral organs of the stamenoid-tepal variety, while extremely low expression of *CsAG1* could be detected in a multi-tepal variety. We further ectopically expressed *CsAG1* in wild type *Arabidopsis*, and observed petal-to-stamen homeotic conversion in several independent transgenic lines, supporting a conserved C-function of CsAG1 in *Cymbidium* flower development. Our results support not only the occurrence of a duplication event during the diversification of Orchidaceae AG lineage, but also a possible link between *CsAG1* and the origin of different floral varieties in *C. sinense*.

## Results

### Flower comparison between the standard and stamenoid-tepal *C. sinense*

We dissected a mature flower from the standard and stamenoid-tepal *C. sinense*, respectively (Fig. 1b, e). The variety with stamenoid tepals in *C. sinense* named “Ling-Nan-Da-Mei” (Csm, Fig. 1d). “Mei” literally in Chinese describes plum-blossom-shaped flower, whose outer tepals become shorter compared with those on the standard *C. sinense* (Fig. 1e). In the standard *C. sinense*, the two inner tepals stretch outward naturally, while the lip forms a coil decorated with different patterns of pigments (Fig. 1b). However, in the stamenoid-tepal variety, those abnormal areas on the inner tepals and lip mimic pollinium structures of the column, forming three inward pockets bending toward the stamen (Fig. 1e). The distal margins of three outer tepals in the stamenoid-tepal variety also become oval and curved compared with the standard *C. sinense* (Fig. 1b, e). We observed the early flower developmental process of the two varieties using scanning electron microscopy (SEM). Although the initiation of floral organ primordia is normal in the stamenoid-tepal variety compared with the standard (data not shown), the same does not occur in later developmental processes. The morphology of the tepals in the stamenoid-tepal variety becomes abnormal, with the whole floral meristem forming an equilateral triangle shape and the top regions of the outer tepals becoming curved, not being able to fully cover the inner floral organs (Fig. 1c, f).

### Isolation and phylogeny of AGAMOUS lineage factors from *C. sinense*

**Fig. 2:**
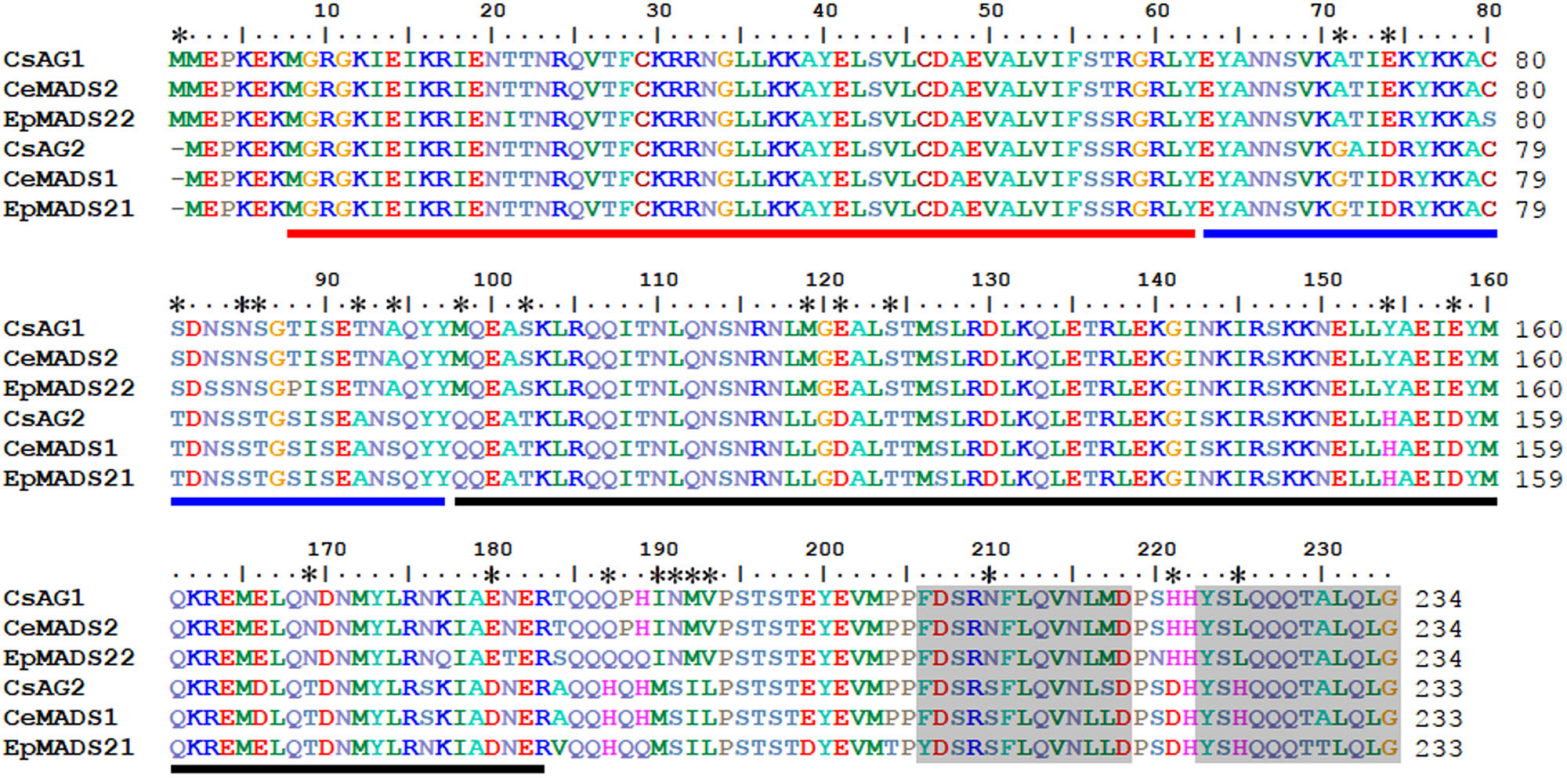
Multiple alignments of different Orchidaceae AGAMOUS-/AG-like proteins. The red, blue and black lines indicate MADS-domain, I-domain and K-domain, respectively; the two AG motifs at the C-terminal are shadowed by gray color; amino acids marked by asterisks indicate polymorphisms between the two sub-clades of orchid AG proteins

**Fig. 3:**
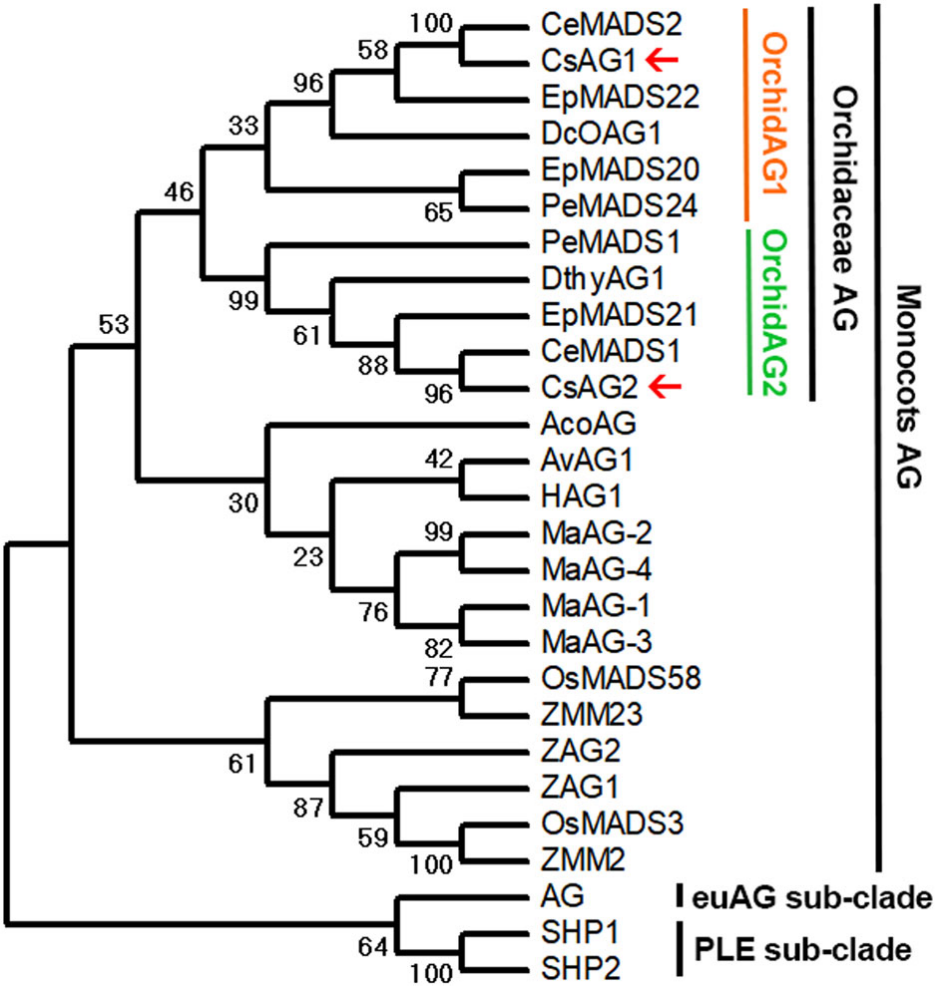
Maximum-likelihood phylogeny tree of AGAMOUS-/AG-like proteins. Proteins from *Arabidopsis* were selected as outgroups; 500 replicates of bootstrap values are marked on each node; the red arrows indicate two AG paralogues isolated from *Cymbidium sinense*

Since genes from the AGAMOUS lineage play a vital role in the determination of plant reproductive organ development, we isolated the cDNA of two *AG*-like factors from the inflorescence of the standard *C. sinense*. *CsAG1* and *CsAG2* encode two putative MADS proteins with 234 and 233 amino acids, respectively (Fig. 2). We downloaded different published sequences, which belongs to AG lineage, from eudicot *Arabidopsis*, the grass family species rice and maize, other monocots species including *Ananas comosus*, *Musa acuminata*, *Asparagus virgatus*, *Hyacinthus orientalis* as well as sequences of different Orchidaceae species. Phylogenetic analysis using maximum-Likelihood method showed that AG lineage factors have undergone multiple duplication events during the diversification of angiosperms (Fig. 3). Consistent with previous studies, the duplication events occurred in eudicots were independent with those happened in monocots (Fig. 3). Similarly, independent duplication events were observed in Poaceae, *M. acuminata* and Orchidaceae evolutionary processes, and there have been two different sub-clades within Orchidaceae AG lineage named OrchidAG1 and OrchidAG2 (Fig. 3). Multiple alignments of the two sub-clades of AG proteins from Orchidaceae revealed three conserved protein domains including MADS-domain, I-domain and K-domain, together with two AG motifs in the C-terminal end (Fig. 2). In addition, we have also detected 25 single amino acid polymorphisms between the two sub-clades of orchid AG proteins (Fig. 2). These results indicated that diverged AG-like factors may have been involved in the regulation of orchid flower development.

### Expression of *AGAMOUS* lineage factors in standard and stamenoid-tepal *C. sinense*

**Fig. 4:**
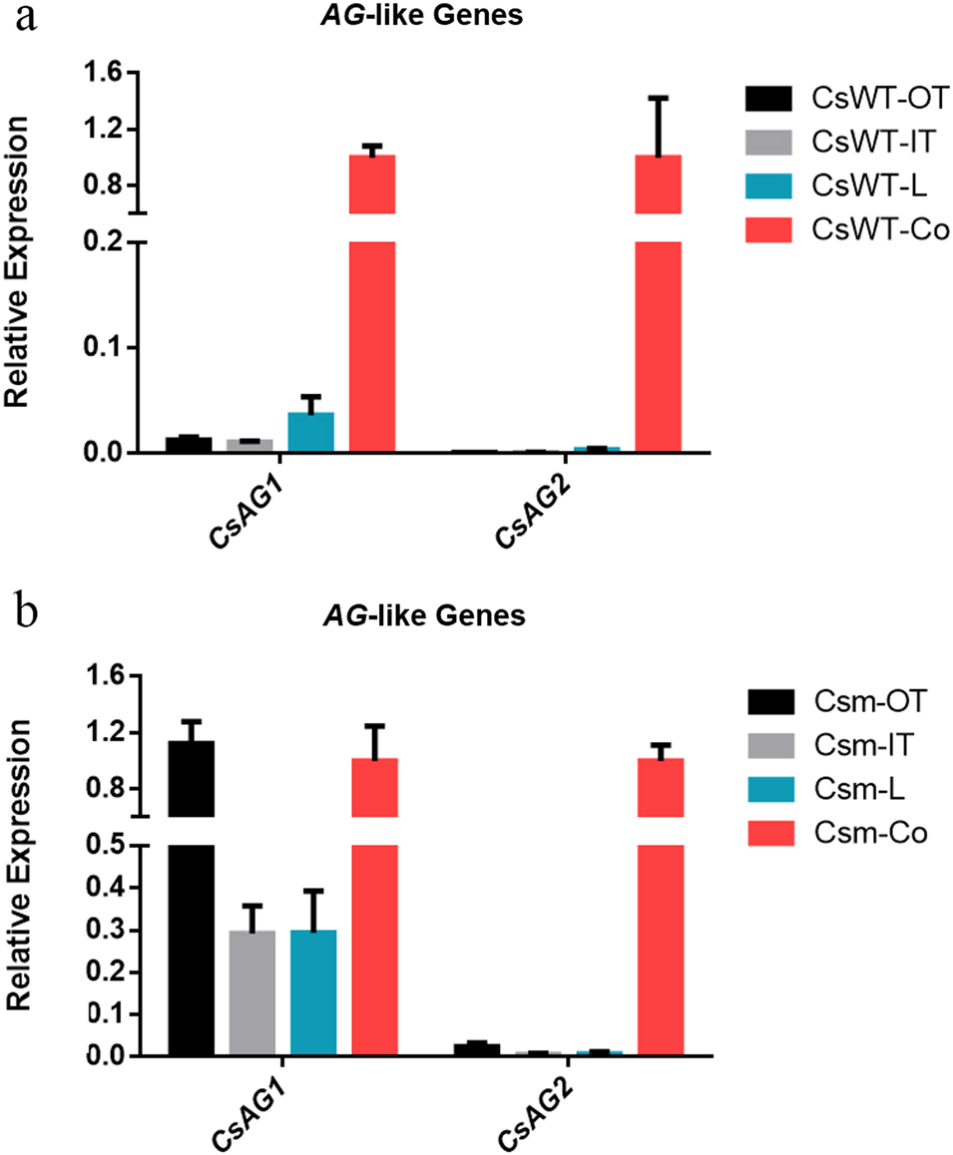
Relative expression of two *AGAMOUS*-/*AG*-like genes in different floral organs of the stage 3 standard (**a**) and stamenoid-tepal (**b**) *Cymbidium sinense*.OT, outer tepal; IT, inner tepal; L, lip; Co, column; the error bar represents the standard deviation of three replicates

We then studied the expression patterns of these two AG lineage genes in different floral organs. Flower buds in different developmental stages were dissected into four parts, including three outer tepals (OT), two inner tepals (IT), a lip (L), and finally, one column (Co). To detect whether the expression of these factors was stage-dependent, we preliminarily examined their expression among three different developing flowers with the bud length of 5 mm (f2), 10 mm (f3), and 15 mm (f4). Although the expression levels of these genes varied in different developmental stages, the overall expression patterns were consistent with the three stages showing high expression in the third column whorl (Fig. 4a, S1). Thus, in later qRT-PCR experiments, we chose f3 stage flowers as materials.

We checked the expression levels of two *AG* paralogues in the stamenoid-tepal variety (Fig. 4b). The results showed that the expression pattern of *CsAG2* was normal compared with the standard *Cymbidium* flower, in which this gene was specifically expressed in the column tissues. Interestingly, ectopic expression of *CsAG1* in outer tepals, inner tepals and lip was detected, being consistent with the stamenoid structures on these floral organs (Fig. 4).

**Fig. 5:**
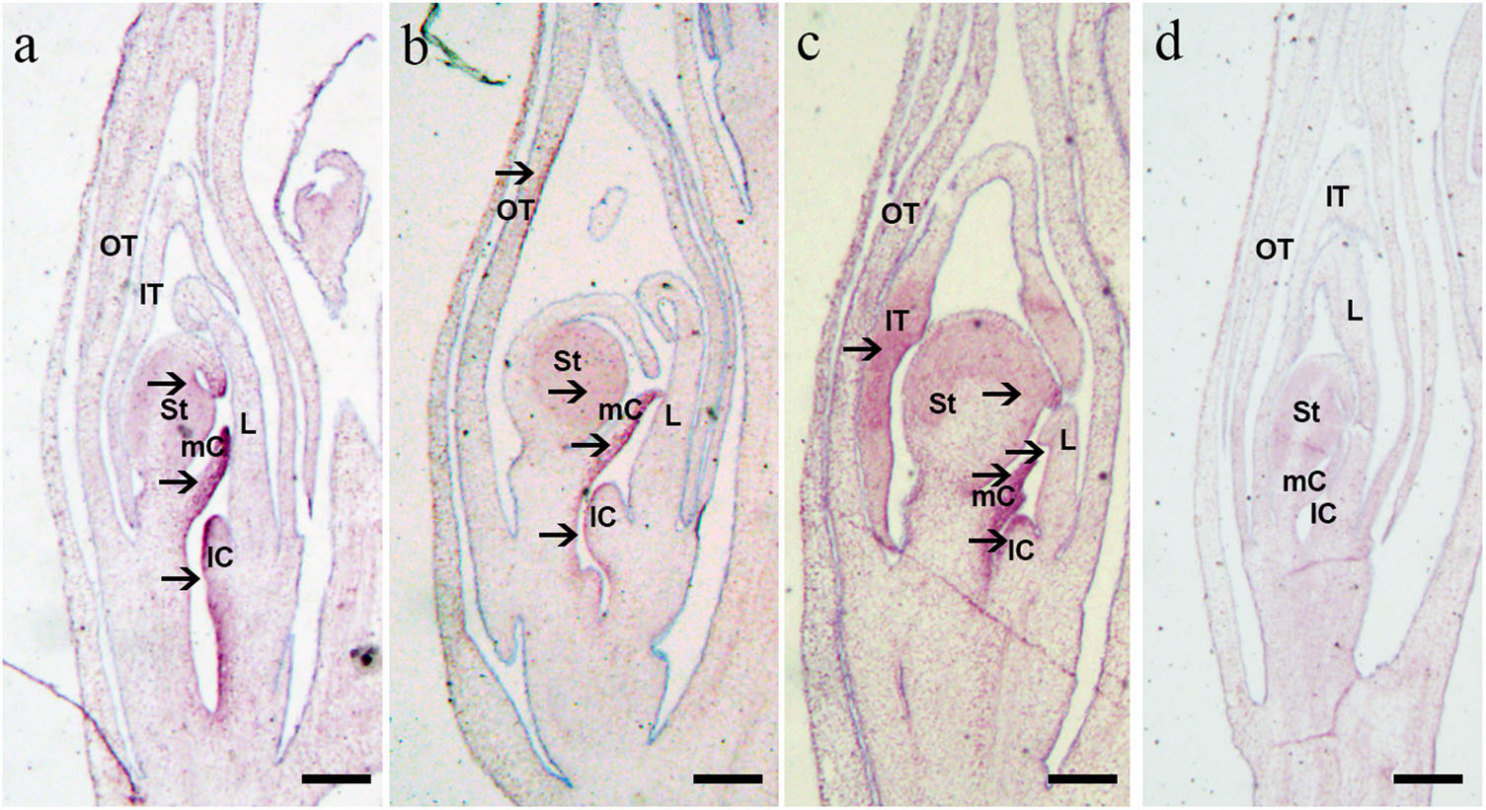
RNA in situ hybridization detected by *CsAG1* antisense probe in standard (**a**) and stamenoid-tepal (**b**, **c**) *Cymbidium sinense*; dark regions marked by black arrows indicate strong signals; (**d**) is a negative control using the sense probe of *CsAG1* in a standard flower; OT, outer tepal; IT, inner tepal; L, lip; St, stamen; mC, median carpel; lC, lateral carpel; bar = 100 μm

We further performed RNA in situ hybridization to check the spatial expression of *CsAG1* (Fig. 5). In the standard *Cymbidium* flower, strong signals could be detected in the stamens and carpels but not the whole column when using anti-sense probe of *CsAG1* (Fig. 5a). The signal was specific since it could not be detected when we used the sense probe (Fig. 5d). Similar with the qRT-PCR assays, ectopic expression of *CsAG1* could be observed in the outer tepal with homeotic conversion in the stamenoid-tepal variety (Fig. 5b). We also detected weak signal in the inner tepal and lip margin from continuous sections of the in situ hybridization assays, which was consistent with the qRT-PCR experiments (Fig. 5c).

### Characterization of another *C. sinense* variety with multiple tepals

**Fig. 6:**
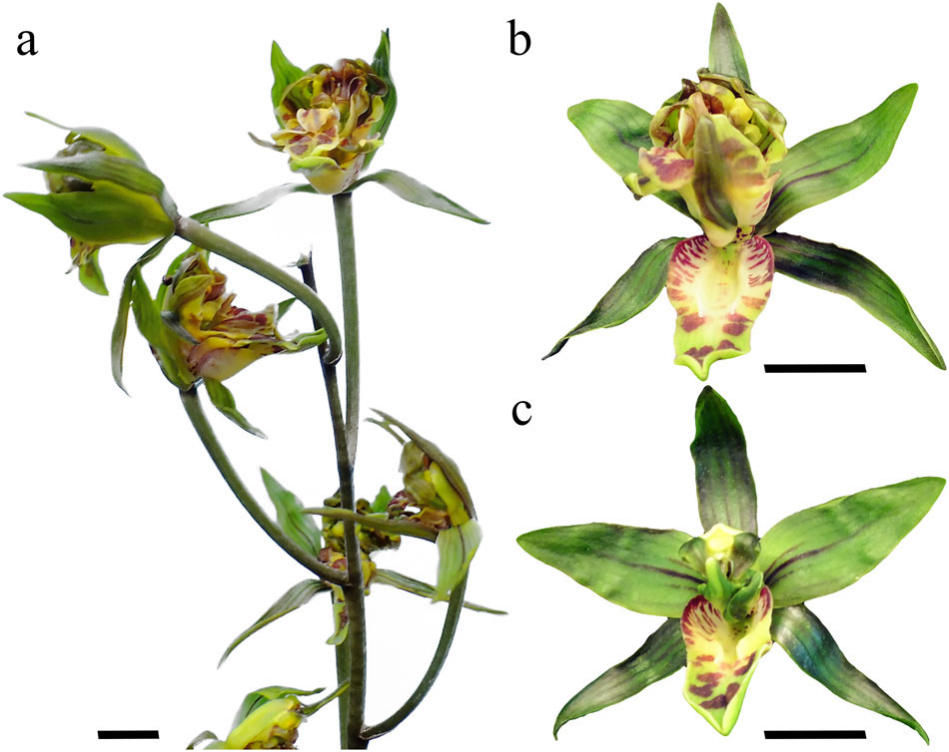
Floral phenotypes of the multi-tepal *Cymbidium sinense*. **a** An inflorescence of the multi-tepal *C. sinense*; (**b**) and (**c**) represent two types of multi-tepal *C. sinense* flowers; bar = 10 mm

In addition, we have collected a variety named Da-Tun-Qi-Lin (Csql) with defects in floral meristem determinacy (Fig. 6). “Qi-Lin”, literally in Chinese, refers to a mythical chimerical creature with fire surrounding its body. Different types of flowers grow along the inflorescence (Fig. 6a). In the severe type, numerous tepals develop in the place of the column (Fig. 6b). In the weak type, several additional tepals surround the abnormal column (Fig. 6c). Remarkably, the identity of three outer tepals, two inner tepals and the lip is not affected in the multi-tepal variety, which is similar with the *ag*-like mutants in eudicots (Fig. 6b, c).

**Fig. 7:**
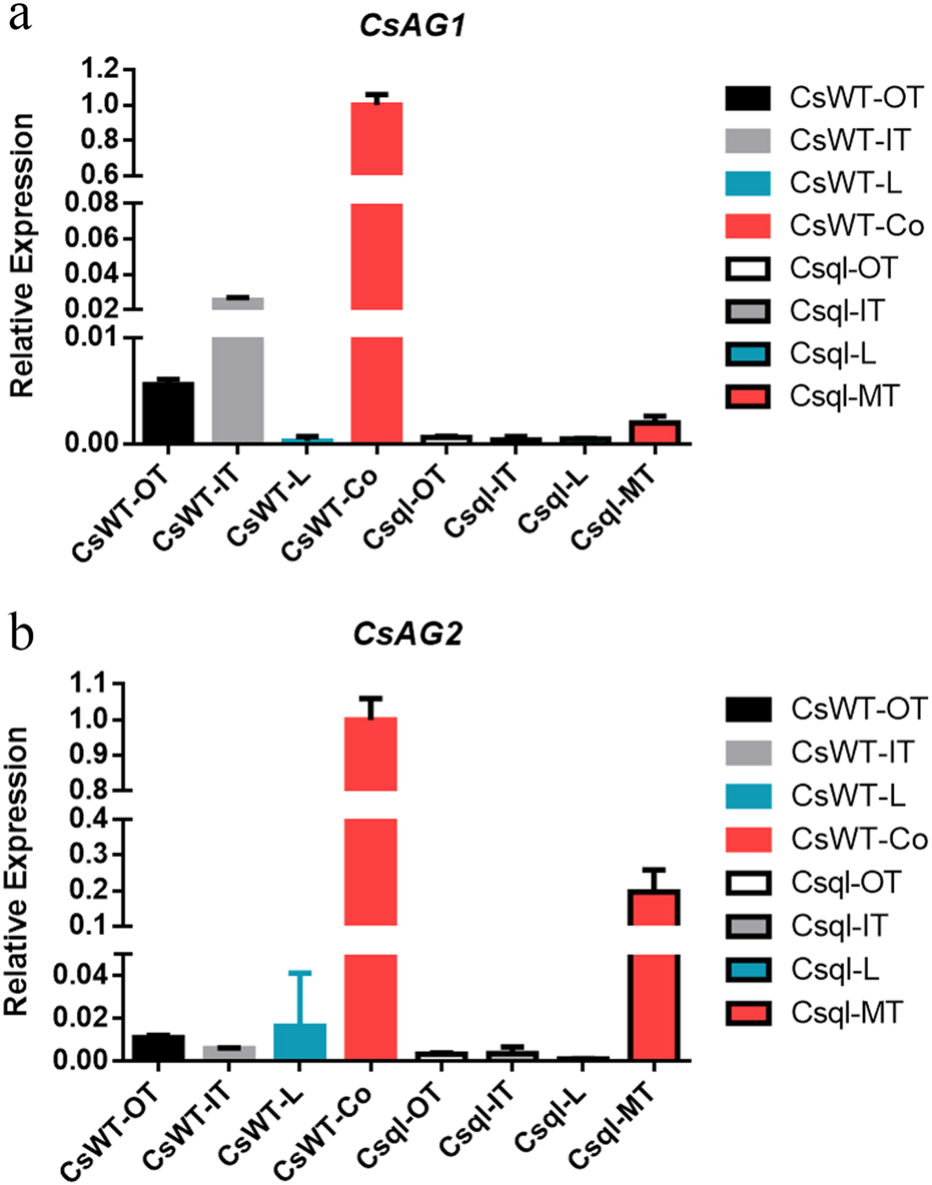
Relative expression of two *AGAMOUS*-/*AG*-like genes, *CsAG1* (a) and *CsAG2* (b), in different floral organs of the stage 3 standard (CsWT) and multi-tepal (Csql) *Cymbidium sinense*.OT outer tepal, IT inner tepal, L lip, Co column, MT multiple inner organs, the error bar represents the standard deviation of three replicates

Thus, we checked the expression of *AG* lineage genes in the multi-tepal variety. Since different degrees of abnormal flowers develop in one plant, we pooled the additional tepals or the abnormal columns, and designated as multi-tepal (MT) to make comparison with the column (Co) tissue in the standard flower. qRT-PCR analysis unraveled that the expression of *CsAG1* and *CsAG2* were all down-regulated in the MT tissue of the multi-tepal variety (Fig. 7). Specifically, the expression of *CsAG1* became extremely low, indicating strong association with the multi-tepal phenotype.

### Phenotypes of 35S:CsAG1 in Arabidopsis

**Fig. 8:**
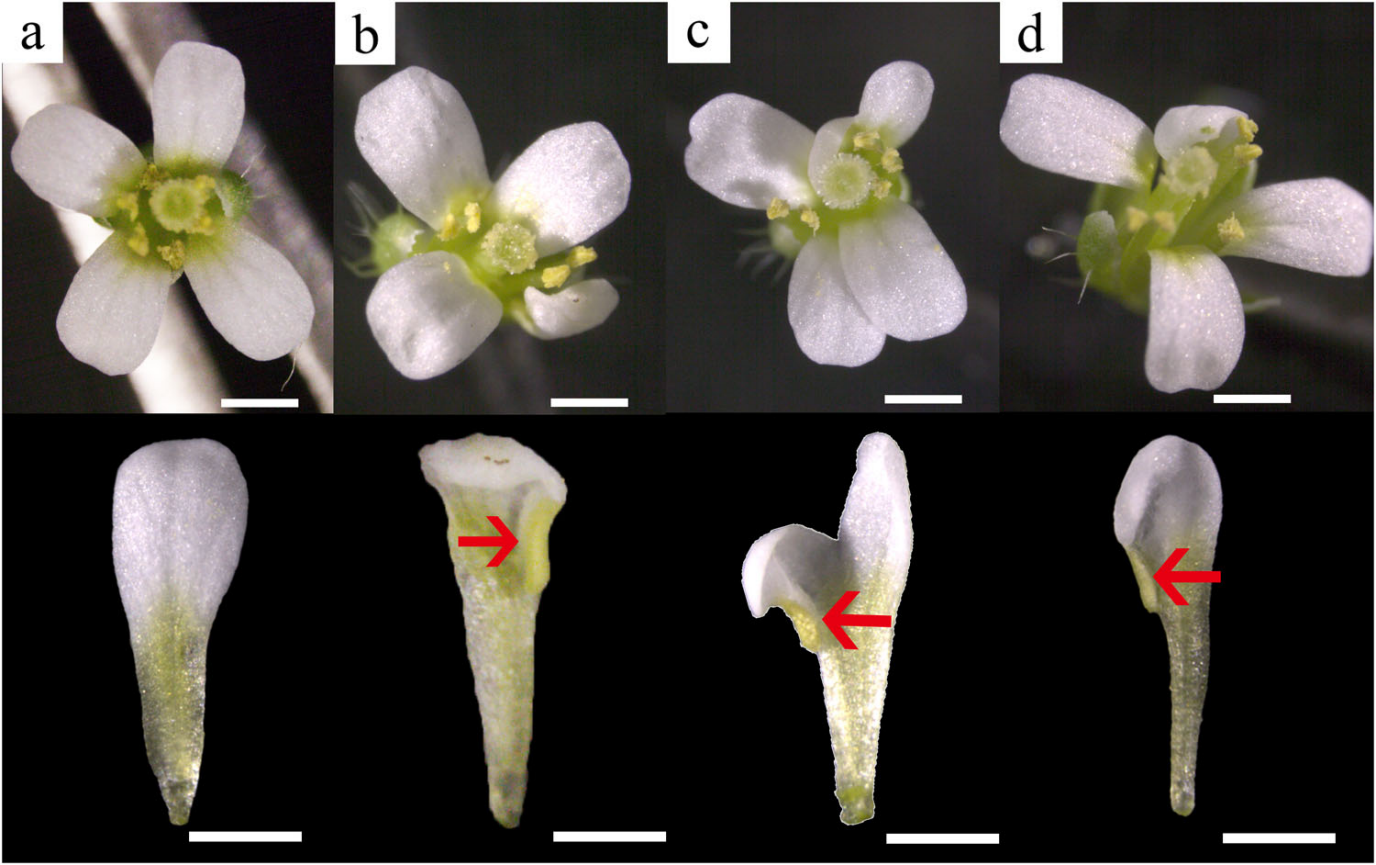
Floral phenotypes of Col-0 (**a**) and three independent lines of *35S:CsAG1* transgenic *Arabidopsis* (**b**-**d**); For each genotype, the upper panel shows an intact flower and the lower panel exhibits a corresponding normal (Col-0) / stamenoid (*35S:CsAG1*) petal; red arrows indicate fused pollen sacs on the stamenoid petals; bar = 2 mm.

Based on the association between phenotype and expression, we inferred a major C function in *Cymbidium* flower that CsAG1 conferred. To verify this hypothesis, we ectopically expressed *CsAG1* in *Arabidopsis* (Fig. 8). In three independent *35* *S:CsAG1* transgenic lines, normal petal identity was disrupted (Fig. 8). Homeotic conversion of petal to stamenoid structure could be detected at the petal margin, which was similar as previous works reported in the eudicots (Fig. 8). These results indicated CsAG1 would possible be a key component with C class function responsible for the orchid reproductive organ development.

## Discussion

Studies in the *AG* lineage genes have been widely conducted in different plant lineages, drawing the conclusion that this gene lineage is associated with floral meristem determinacy and reproductive organ development^5,7,9,11,12,18,20^. *AG* lineage has undergone multiple events of duplication in both monocots and eudicots, leading to possible subfunctionalization^6,10,27,28^. The evolutionary scenario of *AG* lineage is clear in core eudicots with one ancient duplication event in lower eudicots and subsequent production of two sub-clades, including *euAG* and *PLE*^6,10^. Recent duplication events have also been observed in each lineage, as in the case of two *AG*-like genes from *Arabidopsis*, *SHP1* and *SHP2*, which evolved distinct function from *AG* after a duplication event^29^.

In monocots, independent duplication events in *AG* lineage have been observed^6,10,27,28^. In the grass family (Poaceae) before the divergence of maize, rye, wheat and rice, a gene duplication event happened producing two paralogous *AG* lineages defined as *ZAG1* and *ZMM2*^10^. After an allotetraploid event occurred in maize approximately 11.4 Mya, two additional maize *AG* paralogous genes produced, designated as *ZAG2* and *ZMM23*^10,30^. Outside Poaceae, for example, predating the divergence of the Zingiberales, there have been at least two clear sub-clades of the *AG* gene resulted from a single duplication event named *ZinAG-1* and *ZinAG-2*^27^. It is of interest that the expression patterns of the two sub-clades *AG* genes vary in different Zingiberales species, indicating possible mechanisms for the evolution of androecial petaloidy in Zingiberales^27^.

In Orchidaceae, there have been two different sub-clades of *AG* lineage factors resulted from a duplication event predating the divergence of Orchidaceae species (Fig. 3). Both of the two *AG*-like factors were highly expressed in columns of a standard *Cymbidium* flower (Fig. S1), and this is consistent as previous work reported in *Erycina pusilla*, indicating functional redundancy of *AG* lineage factors in Orchidaceae^27^. In this study, we detected the spatial expression pattern of *CsAG1* using RNA in situ hybridization, providing a more detailed pattern in top stamen margin, median carpel as well as the stigma lobe (Fig. 5). We neither detected any signal of *CsAG1* in the floral meristem, nor in the early flower developmental stages, different from the expression of an Orchid*AG2* sub-clade member *PeMADS1* in *Phalaenopsis equestris*, which is abundant in the whole floral meristem^26^. Future work using in situ hybridization assays will allow a precise examination of the expression patterns of different *AG*-like factors, leading to a better understanding under subfunctionalization among these different paralogues.

In the flowers of stamenoid-tepal *C. sinense* variety, although homeotic conversion occurred on each tepal’s marginal regions, the inner floral organs show a severe phenotype comparing to the outer floral organs, despite the fact that higher ectopic expression of *CsAG1* was detected in the outer tepals when compared with the inner tepals and lips (Fig. 1). One possible explanation is the formation of different protein complexes. In *Arabidopsis*, MADS genes determine the floral organ identity through a combinatory way named the ABCDE model^31,32^. Different MADS proteins can form different quaternary protein complexes and bind to the DNA region called CArG-box to regulate the expression of their downstream genes targets^31,32^. In Orchidaceae, previous studies reported that two duplication events happened in the AP3 sub-clade MADS factors, leading to divergence in expression patterns and probably resulting to functional diversification^33–35^. The *AP3-3* and *AP3-4* sub-clades members are highly expressed in the two inner floral organ whorls, while the transcripts of *AP3-1* and *AP3-2* are abundant in the outer tepals and inner tepals, which may result in the formation of different MADS complexes in the stamenoid-tepal *C. sinense* variety^33–35^.

In *Cymbidium ensifolium*, an Orchidaceae *AG2* sub-clade member, *CeMADS1*, is not expressed in *multitepal* flower buds^24^. Unlike the multi-tepal variety of *C. sinense* in this study, the *multitepal* mutants of *C. ensifolium* completely lose the column, together with abnormalities developed in the inner tepals and the lip^24^. Since different *Cymbidium* species have been bred independently, it is possible that different mutations occurred during the generation of these varieties, which favor potential functional divergence within the two clades of AG proteins. A previous study found that complex autoregulatory networks of MADS proteins exist during the floral development of *Arabidopsis*^36^. Another possible explanation for generation of the multi-tepal phenotype in *C. sinense* would be a combinatory manner of two *AG*-like genes, since both of which were down-regulated in the multi-tepal variety.

Column, also known as gynostemium, is a very exquisite structure which has attracted many naturalists since 19th century. Charles Darwin noticed that the specialized structures orchid flower reflects the beauty of the adaptations^37^. A mature column consists of anther, lateral and ventral gynostemium appendages, ovary and calyculus, rostellum and stigma lobes, column-part as well as column-foot^38–40^. Although the morphologically developmental processes of the column have been well described, the underlying molecular mechanisms controlling the structure differentiation remain unclear. Since numerous varieties exist in the *Cymbidium* genus, it would be a choice to use these varieties to study the dark matter behind the orchid flower. Due to the developmental novelties in Orchidaceae flower, it will be exciting to utilize a model system, such as *Erycina pusilla*, for functional studies in the future^41^.

## Materials and Methods

### Plant materials and nucleic acid extraction

All the cultivated *Cymbidium* varieties analyzed in this paper were kept in the greenhouse of National Orchid Conservation Center of China and Orchid Conservation and Research Center of Shenzhen, Shenzhen, China. The *Arabidopsis* were grown in growth chambers at 22 °C under a 16 h of light/8 h dark with 70–80% relative humidity. The *Arabidopsis* and *Cymbidium* genomic DNA was extracted from juvenile leaves by DNA extraction solution containing 2% CTAB. Plant total RNA was extracted from different tissues using Plant RNA Kit (Omega Bio-Tek, Guangzhou, China).

### Scanning electron microscopy

Five centimeter inflorescences were collected for SEM observation. To generate the epoxy replica, the unnecessary tissues were quickly removed and the inflorescences were dissected. The 1st type of impression material (Coltene Ltd. PRESIDENT light body, Art. No. 4667, Switzerland) was daubed onto the surface of dissected inflorescences, and the epoxy mold was fixed upside down on the 2nd type of vinyl polysiloxane impression material (Imprint™ II Garant, 3 M ESPE, U. S. A.). To fill the mold with the 3rd type of epoxy adhesive (Devcon Ltd. 2-Ton Epoxy, Ireland), the inflorescences were removed thoroughly under a stereomicroscope and were kept into 37 °C incubator overnight. The epoxy replicas were sputtered with gold and observed under a JEOL JSM 6360LV Scanning Electron Microscope. The photo contrast was adjusted by using Adobe Photoshop CS6 (Adobe, San Jose, CA, USA).

### Molecular cloning and phylogenetic analysis

To get completed sequences of *AG*-like genes, gene specific primers were designed in 5ʹ-/3ʹ-UTR regions and amplification were carried out using genomic DNA and 5 cm inflorescence cDNA as templates, respectively. PCR products were cloned into pMD19-T vector (Takara) before sequencing. The primers sequences used for molecular cloning were listed in the Supporting Information (Table S1). To perform phylogenetic analysis, putative coding region of each gene was predicted by NCBI ORF-Finder (https://www.ncbi.nlm.nih.gov/orffinder/). The nucleotide sequences were further translated in amino acid sequences prior to multiple alignment using MEGA6, and the aligned sequences were further processed to generate Maximun Likelihood tree under 500 of bootstrap replicates^42^.

### Quantitative RT-PCR

A total of 0.5 μg RNA was reversely transcribed and the PCR assays were performed as we previously reported^43^. All the data were normalized against the expression of reference gene ACTIN, as previously described^44^. The transcript levels for these genes were summarized from three replicates. All the primers sequences used in qPCR were listed in the Supporting Information.

### RNA in situ hybridization

Five centimeter inflorescences were collected for in situ hybridization. After removing unnecessary tissues and large flower buds, the dissected inflorescences were fixed overnight in 4% (wt/vol) paraformaldehyde buffer (pH 7.0), which were further embedded with Paraplast (Sigma-Aldrich China, Shanghai, China). The digoxigenin-labeled probes were made and the hybridization processes were carried out as described^45^. All the primers sequences used in RNA in situ hybridization were listed in the Supporting Information.

### Plant transformation

The coding region of *CsAG1* was cloned and inserted into the multiple clone site of a binary vector pCAMBIA1302, which was further transformed into the *Agrobacterium tumefaciens* strains. The *Agrobacterium*-mediated plant transformation was carried out using the floral dipping method as previously described^46^. Seeds of the *35S:CsAG1* transgenic plants were germinated and selected on Murashige and Skoog (MS) culture media containing Hygromycin B (Roche, Shanghai, China).

### Accession numbers

The accession numbers of sequences used for phylogeny from GenBank (https://www.ncbi.nlm.nih.gov/genbank/) or Phytozome 12 (https://phytozome.jgi.doe.gov/) are as follows: AG (X53579); SHP1 (M55550); SHP2 (M55553); OsMADS3 (L37528); OsMADS58 (AB232157); ZAG1 (L18924); ZMM1 (X81200); ZMM23 (AJ430637); EpMADS20 (KJ002745); EpMADS21 (KJ002746); EpMADS22 (KJ002747); DcOAG1 (DQ119840); DthyrAG1 (DQ017702); CeMADS1 (GU123626); CeMADS2 (GU123627); AcoAG (Aco009993); AvAG1 (BAD18011.1); HAG1 (AAD19360.2); MaAG-1 (GSMUA_Achr10G21480_001); MaAG-2 (GSMUA_Achr10G14160_001); MaAG-3 (GSMUA_Achr5T06590_001); MaAG-4 (GSMUA_Achr6G14760_001); PeMADS1 and PeMADS24 were obtained from the online OrchidBase website (http://orchidbase.itps.ncku.edu.tw/). Gene sequences cloned in this study have been deposited into Genbank database with the accession numbers MG021184 and MG021185.

## Electronic supplementary material

SUPPLEMENTAL MATERIAL

## References

[1] Li X (2014). Genetic diversity, population structure, pollen morphology and cross-compatibility among Chinese Cymbidiums. Plant Breed..

[2] Liu, Z., Chen, S., Ru, Z. & Chen, L. *The Genus Cymbidium in China*. (Science Press, Beijing, China, 2006).

[3] Duttke S, Zoulias N, Kim M (2012). Mutant flower morphologies in the wind orchid, a novel orchid model species. Plant Physiol..

[4] Schmidt RJ (1993). Identification and molecular characterization of *ZAG1*, the maize homolog of the *Arabidopsis* floral homeotic gene *AGAMOUS*. Plant Cell.

[5] Yamaguchi T (2006). Functional diversification of the two C-class MADS box genes *OSMADS3* and *OSMADS58* in *Oryza sativa*. Plant Cell.

[6] Dreni L, Kater MM (2014). MADS reloaded: evolution of the *AGAMOUS* subfamily genes. New Phytol..

[7] Yanofsky MF (1990). The protein encoded by the *Arabidopsis* homeotic gene *AGAMOUS* resembles transcription factors. Nature.

[8] Drews GN, Bowman JL, Meyerowitz EM (1991). Negative regulation of the *Arabidopsis* homeotic gene *AGAMOUS* by the *APETALA2* product. Cell.

[9] Bowman JL, Smyth DR, Meyerowitz EM (1991). Genetic interactions among floral homeotic genes of *Arabidopsis*. Development.

[10] Kramer EM, Jaramillo MA, Di Stilio VS (2004). Patterns of gene duplication and functional evolution during the diversification of the *AGAMOUS* subfamily of MADS box genes in angiosperms. Genetics.

[11] Heijmans K (2012). Redefining C and D in the petunia ABC. Plant Cell.

[12] Davies B (1999). *PLENA* and *FARINELLI*: redundancy and regulatory interactions between two *Antirrhinum* MADS-box factors controlling flower development. Embo J..

[13] Bowman JL, Smyth DR, Meyerowitz EM (1989). Genes directing flower development in Arabidopsis. Plant Cell.

[14] Huang Z (2017). APETALA2 antagonizes the transcriptional activity of *AGAMOUS* in regulating floral stem cells in *Arabidopsis thaliana*. New Phytol..

[15] Pinyopich A (2003). Assessing the redundancy of MADS-box genes during carpel and ovule development. Nature.

[16] Causier B (2005). Evolution in action: following function in duplicated floral homeotic genes. Curr. Biol..

[17] Dreni L (2011). Functional analysis of all *AGAMOUS* subfamily members in rice reveals their roles in reproductive organ identity determination and meristem determinacy. Plant Cell.

[18] Mena M (1996). Diversification of C-function activity in maize flower development. Science.

[19] Theißen G, Strater T, Fischer A, Saedler H (1995). Structural characterization, chromosomal localization and phylogenetic evaluation of two pairs of *AGAMOUS*-like MADS-box genes from maize. Gene.

[20] Ambrose BA (2000). Molecular and genetic analyses of the*silky1* gene reveal conservation in floral organ specification between eudicots and monocots. Mol. Cell.

[21] Lin C (2016). Transcriptome-wide analysis of the MADS-box gene family in the orchid *Erycina pusilla*. Plant Biotechnol. J..

[22] Salemme M, Sica M, Gaudio L, Aceto S (2013). The *OitaAG* and *OitaSTK* genes of the orchid *Orchis italica*: a comparative analysis with other C- and D-class MADS-box genes. Mol. Biol. Rep..

[23] Xu Y (2006). Floral organ identity genes in the orchid *Dendrobium crumenatum*. Plant J..

[24] Wang S (2011). Duplicated C-Class MADS-box genes reveal distinct roles in gynostemium development in *Cymbidium ensifolium* (Orchidaceae). Plant Cell Physiol..

[25] Hsu HF (2010). C/D class MADS-box genes from two monocots, orchid *(Oncidiu*mGower Ramsey) and lily (*Lilium longiflorum*), exhibit different effects on floral transition and formation in *Arabidopsis thaliana*. Plant Cell Physiol..

[26] Chen Y (2012). C- and D-class MADS-box genes from *Phalaenopsis equestris* (Orchidaceae) display functions in gynostemium and ovule development. Plant Cell Physiol..

[27] Almeida AM, Yockteng R, Otoni WC, Specht CD (2015). Positive selection on the K domain of the AGAMOUS protein in the Zingiberales suggests a mechanism for the evolution of androecial morphology. Evodevo.

[28] Zahn LM (2006). Conservation and divergence in the *AGAMOUS* subfamily of MADS-box genes: evidence of independent sub- and neofunctionalization events. Evol. Dev..

[29] Liljegren SJ (2000). *SHATTERPROOF* MADS-box genes control seed dispersal in *Arabidopsis*. Nature.

[30] Gaut BS, Doebley JF (1997). DNA sequence evidence for the segmental allotetraploid origin of maize. Proc. Natl Acad. Sci. USA.

[31] Pelaz S (2000). B and C floral organ identity functions require *SEPALLATA* MADS-box genes. Nature.

[32] Coen ES, Meyerowitz EM (1991). The war of the whorls: genetic interactions controlling flower development. Nature.

[33] Mondragón-Palomino M, Theißen G (2011). Conserved differential expression of paralogous *DEFICIENS*- and *GLOBOSA*-like MADS-box genes in the flowers of Orchidaceae: refining the ‘orchid code’. Plant J..

[34] Aceto S, Gaudio L (2011). The MADS and the beauty: genes involved in the development of orchid flowers. Curr. Genom..

[35] Chang YY (2010). Characterization of the possible roles for B class MADS box genes in regulation of perianth formation in orchid. Plant Physiol..

[36] Kaufmann K (2009). Target genes of the MADS transcription factor SEPALLATA3: integration of developmental and hormonal pathways in the Arabidopsis flower. PLoS Biol..

[37] Darwin C (1862). On the Various Contrivances by Which British and Foreign Orchids are Fertilised by Insects.

[38] Kurzweil H (1987). Developmental studies in orchid flowers I: epidendroid and vandoid species. Nord J. Bot..

[39] Kurzweil H (1988). Developmental studies in orchid flowers III: Neottioid species. Nord J. Bot..

[40] Kurzweil H (1987). Developmental studies in orchid flowers II: Orchidoid species. Nord J. Bot..

[41] Lee S (2015). Establishment of an Agrobacterium-mediated genetic transformation procedure for the experimental model orchid *Erycina pusilla*. Plant Cell Tiss. Org..

[42] Tamura K (2013). MEGA6: molecular evolutionary genetics analysis version 6.0. Mol. Biol. Evol..

[43] Su S (2017). The CYCLOIDEA-RADIALIS module regulates petal shape and pigmentation, leading to bilateral corolla symmetry in *Torenia fournieri* (Linderniaceae). New Phytol..

[44] Zhu G (2015). Transcriptome characterization of *Cymbidium sinense* ‘Dharma’ using 454 pyrosequencing and its application in the identification of genes associated with leaf color variation. Plos One.

[45] Coen ES (1990). *Floricaula*: a homeotic gene required for flower development in *Antirrhinum majus*. Cell.

[46] Clough SJ, Bent AF (1998). Floral dip: a simplified method for Agrobacterium-mediated transformation of *Arabidopsis thaliana*. Plant J..

