# Supplementary material for: An AGAMOUS-like factor is associated with the origin of two domesticated varieties in Cymbidium sinense (Orchidaceae)
Source: Hortic Res. 2018 Sep 1;5:48. doi: 10.1038/s41438-018-0052-z (PMC6119200; doi:10.1038/s41438-018-0052-z)
Supplement: Supplementary file 1 — SUPPLEMENTAL MATERIAL [file 41438_2018_52_MOESM1_ESM.docx]

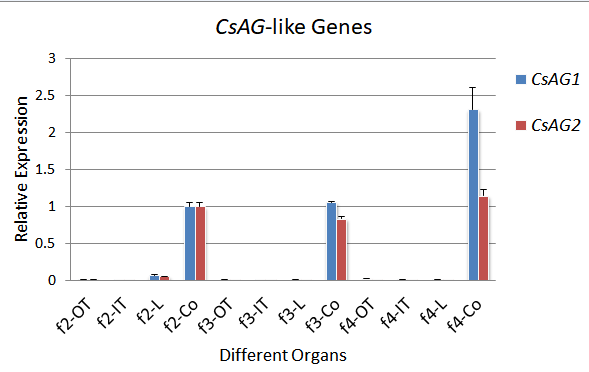


**Fig. S1** Relative expression of two *AGAMOUS*-/*AG*-like genes in different floral organs of the standard *Cymbidium sinense* in different developmental stages; f2: stage 2 flower buds (5 ± 1 mm); f3: stage 3 flower buds (10 ± 1.5 mm); f4: stage 4 flower buds (15 ± 2 mm); OT, outer tepal; IT, inner tepal; L, lip; Co, column; the error bar represents the standard deviation of three replicates.

| Table S1 Primers used in this study | |  |
| --- | --- | --- |
| Primers | Sequences(5'-3') | Proposes |
| CsAG1-qF1 | GTCCTCTGCGATGCTGAAGTC | qRT-PCR |
| CsAG1-qR1 | ATAGTTGCCTTCACACTGTTATTGG | qRT-PCR |
| CsAG2-qF1 | AAACTGATAACATGTACCTGCGTAGTAAG | qRT-PCR |
| CsAG2-qR1 | TGTTGATGGCAGTATGCTCATATG | qRT-PCR |
| CsACT-qF1 | TGCCATGTATGTTGCCATTCA | qRT-PCR |
| CsACT-qR1 | CACCAGAATCCAGCACAATACC | qRT-PCR |
| CsAG1-is-F1 | AGTCCTCTGCGATGCTGAAG | *in-situ* |
| CsAG1-is-R1 | TGGCTGCTGTTGCGTTCT | *in-situ* |
| CsAG1-F1 | ATGATGGAGCCAAAGGAAAAG | CsAG1 cloning |
| CsAG1-R1 | ACCTAATTGGAGGGCAGTC | CsAG1 cloning |
| CsAG2-F1 | ATGGAGCCCAAGGAGAAGATG | CsAG2 cloning |
| CsAG2-R1 | CCCAAGCTGCAGAGCAGTCT | CsAG2 cloning |
